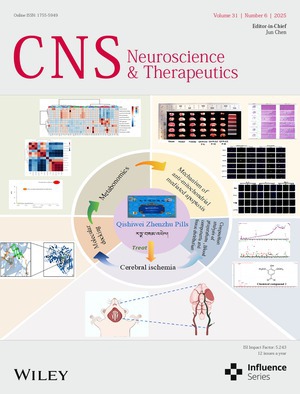# Front Cover

**DOI:** 10.1111/cns.70498

**Published:** 2025-06-29

## Abstract

The cover image is based on the article *Single‐Cell RNA Sequencing and Spatial Transcriptomics Reveal a Novel Mechanism of Oligodendrocyte–Neuron Interaction in Cognitive Decline After High‐Altitude Cerebral Edema* by Wenying Lv et al., https://doi.org/10.1111/cns.70485.